# Carotenoid-based coloration in cichlid fishes

**DOI:** 10.1016/j.cbpa.2014.03.006

**Published:** 2014-07

**Authors:** Kristina M. Sefc, Alexandria C. Brown, Ethan D. Clotfelter

**Affiliations:** a Institute of Zoology, University of Graz, Universitätsplatz 2, 8010 Graz, Austria; b Department of Biology, Amherst College, Amherst, MA 01002, USA; c Graduate Program in Organismic and Evolutionary Biology, University of Massachusetts, Amherst, MA 01003 USA

**Keywords:** Pigment, Trade-off, Antioxidant, Signal, Cichlidae

## Abstract

Animal colors play important roles in communication, ecological interactions and speciation. Carotenoid pigments are responsible for many yellow, orange and red hues in animals. Whereas extensive knowledge on the proximate mechanisms underlying carotenoid coloration in birds has led to testable hypotheses on avian color evolution and signaling, much less is known about the expression of carotenoid coloration in fishes. Here, we promote cichlid fishes (Perciformes: Cichlidae) as a system in which to study the physiological and evolutionary significance of carotenoids. Cichlids include some of the best examples of adaptive radiation and color pattern diversification in vertebrates. In this paper, we examine fitness correlates of carotenoid pigmentation in cichlids and review hypotheses regarding the signal content of carotenoid-based ornaments. Carotenoid-based coloration is influenced by diet and body condition and is positively related to mating success and social dominance. Gaps in our knowledge are discussed in the last part of this review, particularly in the understanding of carotenoid metabolism pathways and the genetics of carotenoid coloration. We suggest that carotenoid metabolism and transport are important proximate mechanisms responsible for individual and population-differences in cichlid coloration that may ultimately contribute to diversification and speciation.

## 1 Introduction

Carotenoids are an important class of pigments in animals. Vertebrates cannot synthesize carotenoids endogenously, but dietary carotenoids derived from photosynthetic organisms are responsible for red, orange and yellow hues of many species, including teleost fishes (Fox and Vevers, 1960; Goodwin, 1984; Kodric-Brown, 1998). In the integument, carotenoid pigments are stored in xanthophores and erythrophores (yellow and red pigment cells, respectively). In fishes and other poikilothermic vertebrates, these types of chromatophores can also synthesize yellow to red pteridine pigments (Braasch et al., 2007). In addition to their role in body coloration, carotenoids have important roles in vision, as precursors of transcription regulators, as antioxidants, and in the immune system (Bendich and Olson, 1989; von Schantz et al., 1999; McGraw and Ardia, 2003; Hill and Johnson, 2012). The size and hue of pigmented tissues are often subject to natural and sexual selection (Grether et al., 2004), and can be important traits in speciation (Boughman, 2002; Maan et al., 2006a). The role of body color in speciation is especially important in animals with acute color vision, such as birds and fishes.

Cichlid fishes (Perciformes: Cichlidae), a group of teleosts endemic to both the Old and New World, provide an excellent system in which to test hypotheses regarding coloration and speciation. Among the Old World cichlids of East Africa, which represent some of the most explosive adaptive radiations among vertebrates (Meyer, 1993; Kocher, 2004), body coloration is inextricably linked with diversification. Color patterns of cichlids diverge in sympatry as well as in allopatry, in response to both natural and sexual selection. In many species, mate choice and dominance interactions are profoundly affected by coloration (Maan and Sefc, 2013), and individuals use dynamic color patterns to communicate motivation and status in social and sexual contexts (Nelissen, 1976; Baerends et al., 1986). Some of these color patterns are carotenoid-based, and in other cases the pigments have yet to be identified. In this review we will (i) briefly introduce the hypotheses that address the signaling function of carotenoid ornaments, (ii) summarize our current understanding of the fitness benefits as well as the biochemical and genetic basis of carotenoid-based coloration in cichlids, and (iii) describe future avenues of research with respect to the role of carotenoids in cichlid coloration and evolution.

## 2 Carotenoid coloration in signaling

The display of carotenoid-based body coloration is costly. It requires the intake of sufficient amounts of carotenoids from the diet, diverts ingested carotenoids away from vital physiological processes to ornamentation, and makes its bearer conspicuous to predators (Endler, 1980; Lozano, 1994). Consequently, carotenoid-based coloration is believed to constitute an honest, condition-dependent signal with functions in both sexual and social contexts (Olson and Owens, 1998; Svensson and Wong, 2011).

Carotenoids are part of the antioxidant arsenal of animals. Antioxidants quench the potentially harmful pro-oxidant molecules generated during normal metabolism. Oxidative stress arises when the balance between antioxidants and pro-oxidants is disturbed, which can be due to a deficiency of antioxidants or to a surplus of pro-oxidants produced by processes such as somatic growth or an immune response. Animals employ a variety of endogenously produced and food-derived antioxidant compounds, including pigments, vitamins, enzymes and other proteins, and the different compounds can interact with and compensate for each other. Apart from their role as antioxidants, carotenoids may also enhance the immune system through increased T-cell activation, macrophage capacity and lymphocyte proliferation (Bendich and Olson, 1989; Pérez-Rodríguez, 2009).

An influential hypothesis regarding carotenoids posits that trade-offs arise from the dual use of carotenoids for physiological functions and ornamentation (Lozano, 1994; von Schantz et al., 1999; Lozano, 2001). The “carotenoid trade-off hypothesis” suggests that competing physiological demands for carotenoid pigments for immunity and oxidative protection may increase the cost of carotenoid allocation to the skin (McGraw and Ardia, 2003; Clotfelter et al., 2007; Peters, 2007; Alonso-Alvarez et al., 2008; Vinkler and Albrecht, 2010; Svensson and Wong, 2011). This scenario requires that carotenoids are in limited supply in the natural diet (Hill, 1992), an assumption that has not found unanimous approval (Hudon, 1994) and lacks empirical evidence (Hill and Johnson, 2012). However, there is considerable support for two predictions of the carotenoid trade-off hypothesis (reviewed in Blount and McGraw, 2008; Svensson and Wong, 2011). The first is that carotenoid supplementation increases coloration, immunity and/or antioxidant capacity (Hill et al., 2002; Blount et al., 2003; Clotfelter et al., 2007). The second is that immune challenges, which also cause oxidative stress, cause re-allocation of carotenoids and reduce coloration of carotenoid-pigmented structures (Perez-Rodriguez et al., 2010; Toomey et al., 2010).

In the past decade, the importance of carotenoids as *in vivo* antioxidants has been questioned (Costantini and Møller, 2008; Pérez-Rodríguez, 2009). Hartley and Kennedy (2004) suggested that carotenoids themselves are particularly vulnerable to oxidative damage, and that carotenoid-based ornamentation is therefore an honest indicator of the antioxidant capacity of other, non-pigment molecules such as vitamin E, rather than itself being traded against antioxidant defense. This hypothesis has become known as the “protection hypothesis” (Pérez et al., 2008) because the non-pigment antioxidants protect the carotenoids from oxidation. A related hypothesis, termed the “sparing hypothesis” (Svensson and Wong, 2011), posits that non-pigment antioxidants may protect carotenoids from oxidation (similar to the protection hypothesis), but that carotenoids are still important components of the antioxidant arsenal. The presence of non-pigment carotenoids (again, such as vitamin E) allows the animal to “spare” carotenoids for re-allocation to other functions such as coloration.

While these hypotheses focus on the availability of, and competition for, antioxidant resources, Hill and Johnson (2012) concentrate on the efficiency of cellular processes, which simultaneously control carotenoid coloration, vitamin A homeostasis and redox balance (“shared pathways,” Hill, 2011). In their view, carotenoid coloration signals body condition by reflecting how well such cellular processes are functioning. Physiological models of shared pathways were developed from bird data (Hill and Johnson, 2012; Johnson and Hill, 2013), and although some metabolic pathways and the tissues in which they occur are different in fish (Katsuyama and Matsuno, 1988), the concept may in principle apply to cichlids and other fishes as well.

Another potential clue to the signal value of carotenoid coloration is its connection to glucocorticoid hormones, which are released during the stress response. Glucocorticoids can increase oxidative stress (Costantini et al., 2008) and cause the reallocation of resources to self-maintenance (Bonier et al., 2009). In spite of this, however, positive correlations between glucocorticoids and redness have been reported in lizards (Fitze et al., 2009), fish (Backström et al., 2014) and birds (McGraw et al., 2011; Fairhurst et al., 2014; Lendvai et al., 2013). However, the relationship between stress hormone levels and carotenoid coloration appears to be condition-dependent, constraining the positive correlation to individuals in good condition (Loiseau et al., 2008; Cote et al., 2010). It is possible that only high-quality animals can tolerate high glucocorticoid levels, and thus advertise this ability through their carotenoid coloration. Additionally, the relationship between glucocorticoids and coloration may depend on the net effect of ornament expression on fitness (Candolin, 2000; Cote et al., 2010). Fairhurst et al. (2014) predict that a correlation between coloration and glucocorticoids will exist only when color signals are key to reproductive success, with its direction dependent on the individual's ability to cope with the energetic demands of ornament production.

The alternatives to the carotenoid trade-off hypothesis are based on the assumption that carotenoids are not the currency in which signaling costs are paid. Experimentally discriminating among the different hypotheses is not easily accomplished. The frequently reported effects of immune challenges and antioxidant supplementation on pigmentation are compatible with the traditional trade-off hypothesis as well as with its more recent alternatives (Svensson and Wong, 2011; Hill and Johnson, 2012). So far, the few studies that unambiguously support or contradict one hypothesis do not converge on a common solution. Several recent reviews (Pérez-Rodríguez, 2009; Metcalfe and Alonso-Alvarez, 2010; Svensson and Wong, 2011; Hill and Johnson, 2012) articulate the need for more experiments specifically designed to differentiate among hypotheses, and for more basic information regarding carotenoid availability in nature and the physiological processes involving carotenoids and their derivatives.

## 3 Carotenoids and fitness in cichlids

In many New and Old World cichlids, carotenoid coloration is correlated with indirect measures of fitness, such as low parasite load, high social status and mate preference. For example, the carotenoid-based red coloration displayed by male *Pundamilia nyererei* in Lake Victoria, Africa, correlates with their natural parasite loads, experimental antibody responses and oxidative stress levels (Maan et al., 2006b; Dijkstra et al., 2007, 2011). Consequently, females may use the carotenoid coloration to size up the health and vigor of their prospective mates and indeed they prefer more red males over less red males (Maan et al., 2004). Color-based female mate choice also underlies reproductive isolation from a sympatric blue-colored species *P. pundamilia* (Seehausen and van Alphen, 1998). Furthermore, male–male aggression biases between the two species are based on body color in a frequency-dependent manner (Dijkstra and Groothuis, 2011). The nuptial coloration of *P. pundamilia* contains substantially fewer carotenoids than that of red *P. nyererei* (Maan et al., 2008). In tests of the carotenoid trade-off hypothesis in these closely related species, the red *P. nyererei* males suffered higher oxidative stress and lower immunity in response to social stress than did the blue *P. pundamilia* males (Dijkstra et al., 2011). In territorial competition under laboratory conditions, however, the red *P. nyererei* males were more aggressive and socially dominant over blue *P. pundamilia* males (Dijkstra et al., 2005, 2006, 2009, 2011). The co-existence of territorial males of the two species in the same microhabitat may be facilitated by a balance between the elevated physiological costs associated with the carotenoid-rich male nuptial coloration of *P. nyererei* and their advantages in intrasexual territorial competition (Dijkstra et al., 2011).

**Fig. 1:**
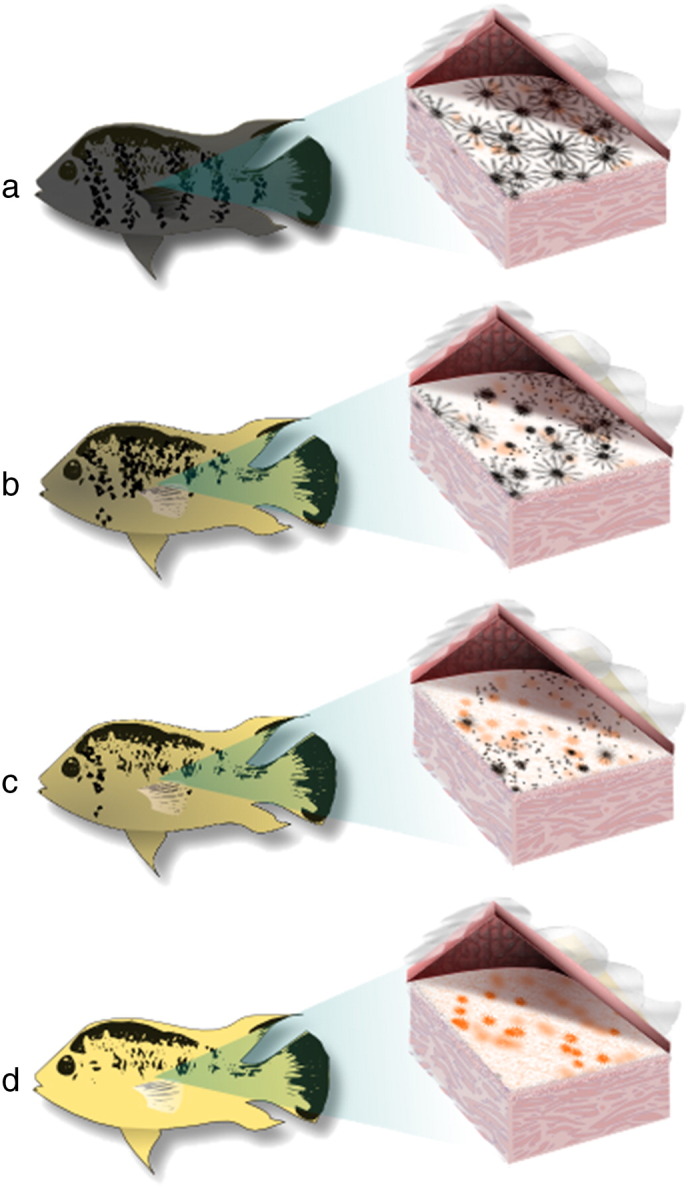
Color polymorphism in the Central American Midas cichlid *Amphilophus citrinellus*. All fish begin life as gray morphs (a). Some fish undergo a color change that involves the dual processes of death of the overlying melanophores and carotenoid deposition in the underlying chromatophores (b–c), resulting in a gold color morph (d). Illustration by Alexandria C. Brown.

Contrary to the findings in *Pundamilia*, the trade-off hypothesis received no support in a New World cichlid, the Midas cichlid *Amphilophus citrinellus*. All Midas cichlids begin life with cryptic gray coloration. As they grow, a minority (8–10%) adopt a distinctive yellow or orange coloration (“gold” morph; Barlow, 1973; Fig. 1). The transition from gray to gold coloration occurs due to the death of the overlying melanophores and accumulation of additional carotenoids in the skin (Dickmann et al., 1988; Fig. 1). Integument carotenoid concentrations, primarily canthaxanthin and tunaxanthin (ε, ε-carotene), are significantly higher in the gold morph than in the gray morph (Webber et al., 1973; Lin et al., 2010). However, contrary to predictions of the carotenoid trade-off hypothesis, dietary supplementation of carotenoids failed to affect skin coloration and did not enhance innate immunity in either morph (Lin et al., 2010). Similarly, findings in female-ornamented convict cichlids *Amatitlania siquia* and *A. nigrofasciata* were contrary to the trade-off, protection and sparing hypotheses. Bacteria-challenged fish experienced reduced oxidative stress while simultaneously allocating more carotenoids to integument, particularly in fish maintained on a trace-carotenoid diet (Brown, 2014). This result suggests that fish can mobilize carotenoids from long-term storage in tissues such as the liver in response to a parasite infection, and calls into question whether carotenoids are limited, which is a central assumption of the carotenoid trade-off hypothesis. Furthermore, subsequent analysis of convict cichlid stomach contents in the field did not support the hypothesis that dietary carotenoids are limited under natural conditions (Brown, 2014).

Social dominance of red or yellow individuals occurs in several cichlid species (Barlow, 1973; Evans and Norris, 1996; Korzan and Fernald, 2007) as well as in other taxa (e.g., Pryke, 2009). Color-based social dominance is apparent in the firemouth cichlid *Thorichthys* (formerly *Cichlasoma*) *meeki*. Fish fed a high-carotenoid diet were more likely to win in dyadic interactions against fish maintained on a low-carotenoid diet (Evans and Norris, 1996). Importantly, this effect disappeared under green light, when the red coloration was no longer visible. Likewise, in *Pundamilia* cichlids, the red male advantage disappeared and staged contests lasted longer when skin pigmentation was obscured by green light (Dijkstra et al., 2005), suggesting that the bright red body color normally has an intimidating effect on blue opponents. The association between carotenoid-based color and social status also occurs in the Midas cichlid. Gold animals are more socially dominant than gray animals. The dominance of gold morphs over equally sized gray morphs results in significantly higher growth rates in gold morphs (Barlow, 1973, 1983). The coloration and larger size of gold morphs inhibit aggression by gray morphs, though gold morphs themselves are not intrinsically more aggressive (Barlow and Wallach, 1976; Barlow and McKaye, 1982). Thus, as in the case of *Thorichthys* (formerly *Cichlasoma*) *meeki* and *Pundamilia*, aggressive advantage may be affected by the perception of carotenoid-based coloration by potential rivals. Dominance of red males also occurs in staged contests between allopatric color morphs of the Lake Tanganyika endemic *Tropheus moorii* (Sefc et al., in review).

Female carotenoid coloration likewise affects competitive interactions (Beeching et al., 1998; Dijkstra et al., 2009). The convict cichlids *A. nigrofasciata* and *A. siquia* (Brown et al., 2013a, 2013b) are reverse-dichromatic; females have a yellow–orange ventrolateral patch that males of this species lack. The function of this carotenoid-pigmented patch is not entirely clear, but laboratory-based experiments suggest that bright coloration incites aggressive responses from other females (Beeching et al., 1998). In a field study, Anderson et al. (in review) observed that female convict cichlids decreased in ventrolateral coloration through the reproductive cycle and with increasing numbers of interactions with predators and heterospecific competitors. Those authors suggest that stress and energy expenditure cause re-absorption of carotenoids from the integument, thus reducing the expression of orange coloration (R.L. Earley, pers. comm.).

Carotenoids have also been detected in the so-called egg spots of several species of Haplochromini and Tropheini cichlids (K.M. Sefc, unpublished data). Egg spots are yellow or orange spots on the anal fins, and are an important synapomorphy in the particularly species-rich haplochromines, a clade of maternal mouthbrooding cichlids comprising the species flocks of Lake Malawi and Lake Victoria (Salzburger et al., 2005). The conspicuousness of the egg spots and their resemblance to the large eggs of the mouthbrooding haplochromine cichlids prompted various hypotheses regarding their role in mate choice, fertilization and reproductive isolation (reviewed in Maan and Sefc, 2013). Recent experiments demonstrated that the effect of egg spots on female choice and male–male aggression varies between species: a female preference for egg spots was detected in *Pseudocrenilabrus multicolor* (Egger et al., 2011), while in *Astatotilapia burtoni* eggs spots affected male–male aggression but not mate choice (Theis et al., 2012). Experiments using computer-animated images revealed a sensory bias for yellow, orange or red spots in female haplochromines, including the most ancestral members of the group (Egger et al., 2011), which could represent a proximate mechanism for the observed effects of egg spots on sexual or social interactions.

## 4 Proximate mechanisms underlying carotenoid coloration

### 4.1 Specification and spatial arrangement of pigment cells

The differentiation of skin pigment cell types from a common precursor, and their migration to and spatial arrangement within the integument, are under genetic control. Zebrafish and medaka mutants have played a major role in the identification of these ‘pigment’ genes (Rawls et al., 2001; Kelsh, 2004; Kelsh et al., 2009; Braasch and Liedtke, 2011; Yamanaka and Kondo, 2014). In zebrafish, for instance, the transcription factor Pax3 is required for the specification of xanthophores, the pigment cells in which carotenoids are stored (Minchin and Hughes, 2008), and xanthophore migration depends on the receptor tyrosine kinase csf1r (Parichy et al., 2000). The expression of the cichlid paralog *csf1ra* in yellow-colored egg spots on the anal fins of Haplochromini cichlids (see below) and in the yellow tips of elongated ventral fins in Ectodini cichlids might be associated with xanthophore recruitment into these tissues (Salzburger et al., 2007). The gene *csf1ra* was also expressed in the yellow areas of the dorsal fin of the cichlids *A. burtoni* and *P. multicolor* (Salzburger et al., 2007). In zebrafish, *csfr1* expressed in xanthophores also contributes to the spatial organization of melanophores in their vicinity (Parichy, 2006), demonstrating how interactions among different chromatophore lineages influence color pattern formation (Kelsh et al., 2009; Yamanaka and Kondo, 2014).

### 4.2 Carotenoid uptake and metabolism

Carotenoids ingested by vertebrates include carotene isomers, which are pure hydrocarbons, and oxygenated carotenoids such as lutein, zeaxanthin and astaxanthin (von Lintig, 2010). Following ingestion, carotenoids are absorbed via diffusion or receptor-mediated transport (von Lintig, 2010; Reboul and Borel, 2011). Dietary lipids may increase carotenoid absorption from the intestinal lumen in mammals (Yonekura and Nagao, 2007), though diet studies in carotenoid-ornamented convict cichlids (Brown, 2014) and lizards (San-Jose et al., 2012) showed that additional lipids decreased body color. Once absorbed, carotenoids are enzymatically modified by conversion or esterification/de-esterification, and transported to the liver and target tissues (e.g. the integument) via lipoprotein transporters. In some fishes, including several cichlids, integumentary carotenoids occur both in free and esterified form (Crozier, 1967; Katsuyama and Matsuno, 1988; Wedekind et al., 1998; Hudon et al., 2003; Lin et al., 2010; Brown et al., 2013b; K. M. Sefc, unpublished data). To date, the types of carotenoids identified in cichlid integument include α- and β-carotene, tunaxanthin, canthaxanthin, astaxanthin, lutein, zeaxanthin, rhodoxanthin, and ‘canary-xanthophyll’ B (Webber et al., 1973; Katsuyama and Matsuno, 1988; Lin et al., 2010; Brown et al., 2013a).

In the aquaculture industry there is considerable interest in the dietary assimilation of carotenoids in economically important fishes, including cichlids cultivated for consumption (e.g. Nile tilapia *Oreochromis niloticus*) and for the ornamental fish trade. Several studies have shown that dietary supplementation increases the integument concentration of carotenoids in cichlids, but effects were not observed in all species and depended on the types of carotenoids that were added (Katsuyama and Matsuno, 1988; Harpaz and Padowicz, 2007; Kop and Durmaz, 2008; Kop et al., 2010; Lin et al., 2010; Güroy et al., 2012; Brown et al., 2013a; Yilmaz et al., 2013). In some cases, the administration of a carotenoid-rich diet also improved growth rates and enhanced reproductive performance (Güroy et al., 2012; Yilmaz et al., 2013; but see Harpaz and Padowicz, 2007; Pan and Chien, 2009). The effects of dietary carotenoids (predominantly astaxanthin) on coloration, growth and performance are consistent with the putative links between carotenoid signals, health and condition. Discrimination between the alternative hypotheses, however, will require further, specifically designed experiments.

**Fig. 2:**
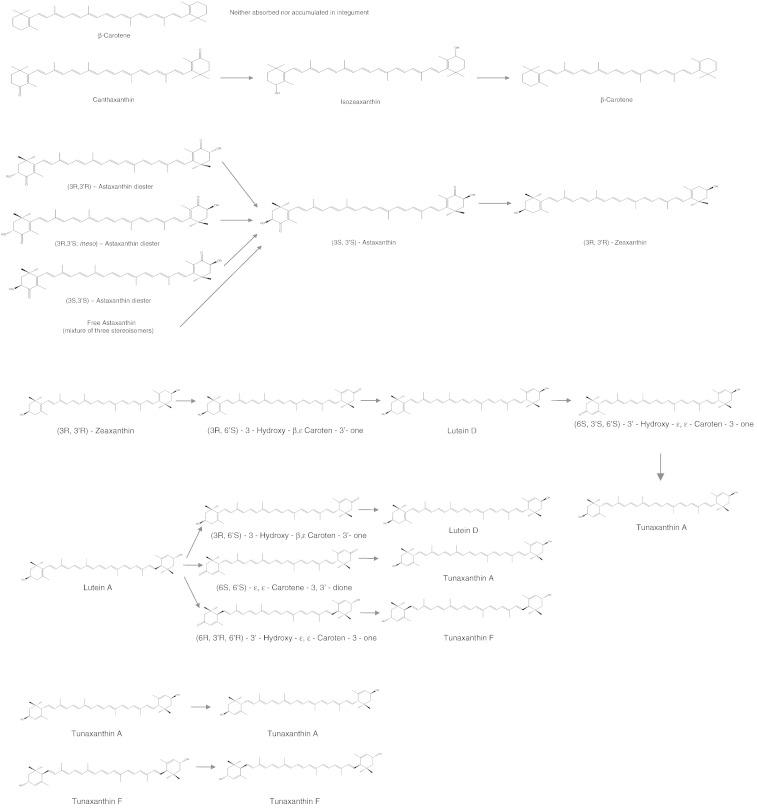
Putative metabolic pathways of carotenoids in the integument of Nile tilapia (*Oreochromis nilotica*). Additional conversion takes place in the liver (not shown). Metabolic pathways in other cichlid species are virtually unknown, highlighting an area in need of further research. Re-drawn with permission from Katsuyama and Matsuno (1988).

Most of what is known about carotenoid metabolism in the cichlid integument comes from feeding experiments with Nile tilapia (Katsuyama et al., 1987; Katsuyama and Matsuno, 1988). The reconstructed metabolic pathways involve epimerization, reduction or oxidation of dietary canthaxanthin, astaxanthin, zeaxanthin and lutein (Fig. 2). Dietary tunaxanthin accumulated in the integument unchanged, whereas dietary β-carotene was neither accumulated nor bioconverted in the integument (Katsuyama and Matsuno, 1988; Fig. 2). It is unclear to what extent the pathways identified in tilapia are conserved across cichlid species. Consistent with the findings in tilapia, dietary β-carotene had smaller effects on body coloration than astaxanthin in feeding experiments with *A. citrinellus* (formerly *Cichlasoma citrinellum*; Pan and Chien, 2009) and *Heros severus* (formerly *Cichlasoma severum*; Kop and Durmaz, 2008). The positive correlation between dietary and integumentary astaxanthin concentrations suggests direct deposition of astaxanthin in *A. citrinellus* (Pan and Chien, 2009).

**Fig. 3:**
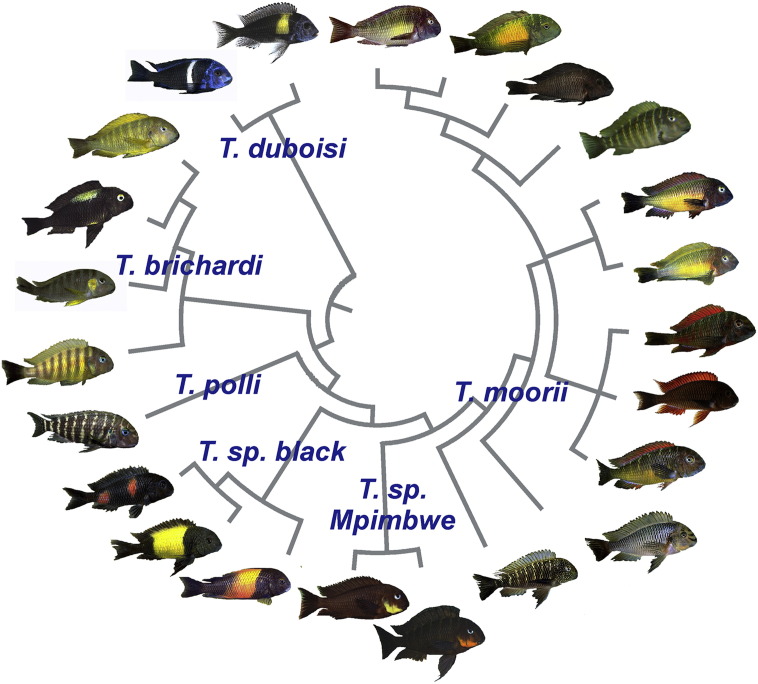
Geographic color variation in a cichlid fish. Phylogenetic relationships among selected examples of the rich variety of differently colored populations of the genus *Tropheus* (Schupke, 2003; Konings, 2013) reveal differentiation in body and fin coloration between close relatives as well as repeated evolution of similar colors. The taxonomy of the genus is not fully resolved; nominal and suggested species supported by genetic data and assortative mating are indicated. The population tree is based on data from Egger et al. (2007) and Koblmüller et al. (2011). Photographs: Ad Konings (*T*. sp. Mpimbwe), Wolfgang Gessl and Peter Berger.

Knowledge of the mechanisms that control the amount and type of carotenoids deposited in integument is prerequisite to understanding the proximate causes of carotenoid color variation. Studies in birds have shown that variation in carotenoid-based coloration can simply reflect presence or absence of carotenoids in the tissue (Eriksson et al., 2008; Walsh et al., 2012), or be caused by differences in carotenoid types (Brush and Seifried, 1968), concentrations (Brush, 1970; Inouye et al., 2001) or both (Crozier, 1967; Hudon et al., 1989). For example, *T. moorii* populations (Fig. 3) differ not only in total carotenoid content but also in the types of integumentary carotenoids, the latter inferred from the shapes of carotenoid absorption spectra and patterns of HPLC chromatograms (Mattersdorfer et al., 2012; K. M. Sefc, unpublished data). Significantly different concentrations of the same types of integumentary carotenoids were found between red and blue *Pundamilia* spp. as well as between the red and yellow body regions of *P. nyererei* (Maan et al., 2008) and between the gold and gray color morphs of the Midas cichlid (Lin et al., 2010). These findings suggest that, as in birds, cichlid carotenoid pigmentation may be determined by different metabolic pathways in different species or populations.

### 4.3 Genetic basis of carotenoid coloration in cichlids

Heritability of carotenoid-based coloration has been demonstrated in different vertebrate species, but to date its genetic basis is only poorly understood (Olsson et al., 2013; Roulin and Ducrest, 2013). Walsh et al. (2012) developed a list of 11 candidate genes with potential roles in the uptake, deposition and degradation of carotenoids in vertebrates (see also Eriksson et al., 2008). Transporter proteins and enzymes involved in the uptake and metabolism of dietary carotenoids are particularly likely to be under genetic, rather than environmental, control (Yonekura and Nagao, 2007; Hill and Johnson, 2012). Genetic factors play a principal role in the generation of discrete variation, such as color polymorphisms or differentiation between closely related cichlid taxa (Magalhaes and Seehausen, 2010; Takahashi et al., 2013; but see Korzan et al., 2008).

The number of genes thus far implicated in cichlid carotenoid coloration is small. Analyses of gene expression in carotenoid-containing integument implicated the chromatophore formation genes *csf1ra* and *Edn3b* as candidate color genes in cichlids (Salzburger et al., 2007; Diepeveen and Salzburger, 2011; see also Section 4.1.). A QTL region for yellow fin coloration identified in Lake Malawi cichlids included neither *csf1ra* nor *Edn3b*, but contained two other candidate carotenoid genes, namely *StAR*, which could play a role in carotenoid binding and deposition, and *BCDO2*, which cleaves carotenoids into colorless metabolites (O'Quin et al., 2013). In the Midas cichlid, inheritance studies have suggested that a single locus is responsible for triggering the transition from gray to gold morph, which involves both melanophore death and carotenoid accumulation (Barlow, 1983; Henning et al., 2010; Fig. 1). Recently, a transcriptomics approach (Henning et al., 2013) identified several differentially expressed genes involved in melanophore maintenance and cell death. In contrast, the genes involved in the upregulation of carotenoid assimilation, transportation and deposition during the color morph transition have yet to be identified.

The orange blotch phenotype of certain Lake Malawi cichlid species, in which females display dark melanophore blotches on a background of xanthophores, was associated with up-regulation of the cichlid *Pax7* gene, a member of the *Pax3/7* subfamily (Roberts et al., 2009). The orange coloration in orange-blotch females is alcohol-soluble and therefore likely to be carotenoid-based (R. Roberts, pers. comm.). In zebrafish, *Pax3* and *Pax7* influence xanthophore specification (*Pax3*) and pigmentation (*Pax7*) as well as melanophore number and size (*Pax3*) (Minchin and Hughes, 2008). Cichlid females carrying the up-regulated allele of *Pax7* (*OB* females) develop fewer but larger melanophores than the brown barred females. *OB* females vary in background color from white to orange depending on species and population, indicating that the identified allele affects the melanophore pattern but not the orange coloration in these cichlids (Roberts et al., 2009). One possible route for cichlid *Pax7* to influence the xanthophore background in orange-blotch cichlids is via developmental trade-offs between decreased melanophore numbers and increased xanthophore numbers (R. Roberts, pers. comm.).

In Midas cichlids and Lake Malawi cichlids with the orange-blotch phenotype, and likely in many other cichlid species, the extent of carotenoid-based coloration on the body is contingent on the distribution of melanophores. This interaction makes the processes controlling melanophore patterning relevant to understanding the processes underlying carotenoid coloration. Studies in zebrafish and medaka provided a wealth of information about the genetics of melanin coloration in these model organisms (Rawls et al., 2001; Kelsh, 2004; Kelsh et al., 2009; Braasch and Liedtke, 2011; Yamanaka and Kondo, 2014). Several candidate melanophore-pattern genes have been studied in cichlids and may be involved in cichlid coloration (Sugie et al., 2004; Diepeveen and Salzburger, 2011; Gunter et al., 2011; O'Quin et al., 2013; but see Watanabe et al., 2007).

## 5 Gaps in our understanding of carotenoid-based coloration in cichlids

Carotenoids are important in many aspects of cichlid biology. The elaboration of carotenoid-pigmented structures via sexual selection is well documented. Much remains to be discovered, however, in areas such as carotenoid biochemistry, physiology and genetics. Basic data on the types and functions of carotenoids in cichlids, and their interactions with other metabolites, will allow the testing of hypotheses regarding the evolutionary significance of carotenoid coloration. Below, we specify some areas for future research that we believe will contribute significantly to our understanding of the evolution of carotenoid pigmentation in cichlid fishes, and in fishes more broadly.

### 5.1 What are the proximate mechanisms that determine, generate and control carotenoid-based coloration in cichlids?

The identification of esterified carotenoids – which are present in the integument of many cichlids – via high-performance liquid chromatography is more complicated than for non-esterified carotenoids, such as in bird feathers. The identification of integumentary carotenoids in cichlids (e.g. Lin et al., 2010) will allow us to assess whether different hues are produced by different types or by different concentrations of carotenoids, and whether similar hues in unrelated taxa result from similar or from taxon-specific mixtures of integumentary carotenoids. Differences in coloration among closely related taxa (e.g. Fig. 3) will make for particularly powerful tools to examine the relationship between carotenoid composition and coloration against similar physiological and ecological backgrounds. Furthermore, such information might allow us to link differences in diet, so important among lacustrine cichlids, to differences in coloration. Combining biochemistry with genome and transcriptome analyses will advance our understanding of the genetics and physiology of carotenoid-based coloration in cichlids and its rapid evolution. Research efforts along these lines will benefit greatly from advances in cichlid genomics (Tilapia Genome Project and Cichlid Genome Consortium, USA). Armed with this knowledge, we can then ask questions about the diversity of metabolic pathways leading towards particular types of carotenoids, and at which phylogenetic levels this variation emerges. Research on other types of chromatophores and pigments, and on structural coloration, will be required to understand and appreciate the interactions that influence the extent and intensity of carotenoid coloration. For example, chromatophore patterns determine the amount of carotenoids to be deposited in the integument, and signal strength can be modified by contrasts between adjacent areas or by overlap of chromatophore layers.

### 5.2 How do carotenoids interact with non-carotenoid pigments and structural colors?

Poikilothermic vertebrates do not rely solely on dietary carotenoids to produce yellow and red coloration; they can also synthesize pterin pigments. Pterins have been detected in the integument of several fish species (Rempeters et al., 1981; Ziegler et al., 2000; Grether et al., 2001). The balance between carotenoid and pterin based pigmentation is intriguing, because pterins theoretically allow the animals to display color largely independent of their diet and health (but see Grether et al., 2004). Interestingly, pterins do not appear to be significant determinants of coloration in the few cichlid species examined so far (*P. nyererei*: Maan et al., 2006b; *T. moorii*: Mattersdorfer et al., 2012; *A. nigrofasciata*: Brown et al., 2013a), but information from additional taxa is sorely needed. There are also unanswered questions regarding the interactions between carotenoids and other pigments such as melanins and structural color components such as iridophores (Brown et al., 2013a; San-Jose et al., 2013; Yamanaka and Kondo, 2014), which can modulate tissue brightness, hue and contrast (Grether et al., 2004).

### 5.3 How do environmental conditions influence carotenoid coloration?

Carotenoid-based coloration of cichlids responds to a number of factors including diet, health status and social stimulation (Katsuyama and Matsuno, 1988; Hofmann and Fernald, 2001; Maan et al., 2006b; Dijkstra et al., 2007; Kop et al., 2010). For example, androgens may be involved in mediating the social stimulation of carotenoid displays, and their possible contributions to the diversity of cichlid behavior and coloration as well as their concomitant impacts on immunity and oxidative status (Kurtz et al., 2007; Peters, 2007; Alonso-Alvarez et al., 2008) certainly merit further attention. As another example, the effects of diet on integumentary carotenoids depend on the availability and functionality of metabolic pathways, and interactions among diet, genotype and physiological state will shed light on proximate and ultimate mechanisms associated with carotenoid coloration (Lin et al., 2010). Finally, the physical environment plays a prominent role in the expression of carotenoid-based coloration. Barlow (1983) noted that the frequency of gold morph Midas cichlids varied among Nicaraguan lakes, and was positively correlated with lake turbidity. The African Rift lakes provide numerous examples of differences in male cichlid nuptial coloration (some of it only putatively carotenoid-based) due to depth, water clarity and substrate type (Seehausen et al., 2008; Maan et al., 2010; Pauers, 2011).

### 5.4 What do carotenoid-based ornaments signal in cichlids?

The conspicuous carotenoid-based coloration of many cichlids is likely to convey information on the bearer's condition, status and motivation. The use of this information in female choice (Maan et al., 2004; Pauers et al., 2004) and male–male competition (Evans and Norris, 1996) has been documented, but is not always obvious (Hermann et al., in review). Although female coloration is common among cichlids, its role in intersexual and intrasexual selection, particularly in reverse sexually-dichromatic species requires further study (Beeching et al., 1998; Tobler, 2007; Baldauf et al., 2011). In addition to clarifying the contexts in which carotenoids signals are used, it is particularly important to understand what information carotenoid signals actually convey. Numerous studies in a wide range of species have revealed correlations between carotenoid coloration and individual physical condition, but no single mechanism has received unequivocal support (Olson and Owens, 1998; Pérez-Rodríguez, 2009; Hill, 2011; Svensson and Wong, 2011). In cichlids, carotenoid signals are employed against the background of very different life histories, including contrasts between territoriality and non-territoriality, between monogamous and polygynous mating systems and between brood care and the absence thereof. It will be illuminating to examine how carotenoid physiology is affected by these life-history differences, and how this in turn affects carotenoid signaling (Fairhurst et al., 2014). Considering the conflicting results between tests of the carotenoid trade-off hypothesis in Old and New World cichlid species, which were supported in the former group but not in the latter, future research should focus on differences in proximate mechanisms in carotenoid conversion and utilization that may have occurred in > 57 million years since these groups diverged (Friedman et al., 2013).

## 6 Why cichlids?

Many of these questions outlined above can be addressed in other organisms, but we believe cichlids are a particularly important group for this kind of research for several reasons. On the one hand, understanding carotenoid physiology and biochemistry is important for our understanding of cichlid evolution. On the other hand, the extraordinary diversity of cichlids will help advance our understanding of the roles of carotenoids in organismal biology.

First, the rich diversity of cichlid color patterns promises opportunities to uncover a variety of genetic, cellular and physiological processes involved in carotenoid coloration and color pattern differentiation (Kocher, 2004). Variation in the extent of sexual dichromatism among related species can inform us about the regulation of color pattern expression (Gunter et al., 2011), as can the status-dependent color changes in the haplochromine cichlid *A. burtoni*, a model species in social neuroscience (Hofmann and Fernald, 2001; Fernald, 2012). Some cichlid species are economically important and the collective interests of the aquaculture industry for improved productivity and flesh coloration can be brought to bear on the subject of evolutionary physiology. The continued development of cichlid genomics, including whole genome sequences, will provide the genetic tools to isolate genes involved in carotenoid absorption, transport and conversion. Testing the function of isolated genes has come within reach with the successful creation of transgenic cichlids (Fujimura and Kocher, 2011; Juntti et al., 2013). The relevance of these findings will certainly extend to other taxa in the conspicuously colorful order of Perciform fishes, and beyond to other species-rich vertebrate groups.

Second, the aforementioned variation in coloration can help us to better understand the relationship between phenotypic plasticity and speciation (Pfennig et al., 2010). Cichlid fishes are textbook cases of adaptive radiation and evolutionary diversification, and we know that color patterns and differences in diet are both important factors involved in speciation in cichlids. For example, assortative mating and social dominance based on carotenoid-based coloration contribute to speciation and species coexistence (Seehausen and van Alphen, 1998; Elmer et al., 2009; Dijkstra and Groothuis, 2011). Moreover, color pattern differences occur at different levels of phylogenetic divergence – polymorphic populations (Fig. 1), intraspecific geographic variation (Fig. 3) and variation across species and tribes – and are sometimes replicated in independent taxon pairs (Maan and Sefc, 2013). We predict that our understanding of this explosive adaptive radiation will be greatly improved if we succeed in expanding our knowledge of the proximate and ultimate mechanisms mediating carotenoid-based coloration, and combine it with the wealth of existing knowledge on cichlid ecology, behavior and phylogenetic relationships.
